# Evaluation of simple antioxidant blood parameters in patients with migraine

**DOI:** 10.3389/fneur.2022.939363

**Published:** 2022-07-26

**Authors:** Zhenzhen Yang, Pengfei Xu, Chaofan Geng, Hongju Zhang

**Affiliations:** ^1^Fuwai Central China Cardiovascular Hospital, Henan Provincial People's Hospital, Zhengzhou, China; ^2^Henan University People's Hospital, Henan Provincial People's Hospital, Zhengzhou, China; ^3^Zhengzhou University People's Hospital, Henan Provincial People's Hospital, Zhengzhou, China

**Keywords:** migraine, uric acid, bilirubin, albumin, creatinine

## Abstract

**Background:**

The study aims to investigate the role of serum albumin (ALB) and creatinine (CRE), bilirubin (BIL), and uric acid (UA) as major intravascular antioxidants in migraine.

**Methods:**

We enrolled 148 patients with migraine and 150 age- and sex-matched healthy controls. The serum levels of ALB, TBIL, CRE, and UA were measured in patients with migraine of different subtypes. The risk of migraine was assessed by multiple stepwise logistic regression analysis.

**Results:**

The serum levels of ALB, total BIL (TBIL), CRE, and UA were significantly lower in the migraine group than in the HC group (*p* < 0.05). The ALB and UA levels were lower during migraine attack periods (*p* < 0.05). There were no statistically significant differences observed in serum ALB, TBIL, CRE, and UA levels between aura/without aura and episodic/chronic migraine subtypes (*p* > 0.05). The multiple stepwise logistic regression revealed that ALB [odds ratio (OR) 0.79, 95% confidence interval (CI) 0.69–0.89, *p* < 0.001], TBIL (OR 0.61, 95% CI 0.5–0.75, *p* < 0.001), and UA (OR 0.97, 95% CI 0.96–0.99, *p* = 0.014) were independently associated with migraine. In addition, the serum levels of ALB, TBIL, and UA were significantly lower in the migraine group when compared by sex.

**Conclusion:**

The serum levels of UA, TBIL, ALB, and CRE were lower in the patients with migraine, indicating a lower antioxidant status. In addition, ALB, TBIL, and UA were independently related to migraine. These results could provide insights into the possible role of oxidative stress in the pathogenesis of migraine.

## Introduction

Migraine is a common primary headache mainly characterized by unilateral or bilateral throbbing headache and special associated features including photophobia, phonophobia, nausea, and vomiting (1), and is estimated to affect 15% of the general population (2). About 30% of patients with migraine perceive aura symptoms, which are a transient symptom in visual disturbances preceding a migraine attack (3). It is widely believed that the trigeminovascular system plays a fundamental role in the pathophysiology of migraine (4). However, the pathophysiology of migraine is not yet fully understood (5). Previous studies have reported that decreased antioxidant levels increasing oxidative stress can lead to vascular inflammation in patients with migraine (6). There is growing evidence supporting an association between neurogenic inflammation and migraine (7). There are relatively few studies on blood markers of antioxidant status in migraine, although it is hypothesized that oxidative stress plays an important role in the pathogenesis of migraine.

More recent studies suggests that serum albumin (ALB), total bilirubin (TBIL), creatinine (CRE), and uric acid (UA) are major non-enzymatic antioxidants in human plasma and can play important antioxidant roles as endogenous reactive oxygen species (ROS) scavengers (8). If these markers can support the involvement of oxidative stress in the pathology of migraine remains unclear. Identification of possible effects of oxidative injury on patients with migraine in the early period may make it possible to contribute to decreasing the frequency of migraine headache attacks and to slowing progression.

On the basis of the above considerations, this study was conducted to investigate the serum levels of ALB, TBIL, CRE, and UA between patients with migraine and healthy controls (HCs). Serum ALB, TBIL, CRE, and UA levels were also investigated in the patients with migraine with different clinical features and subtypes, aiming to determine if any of these markers are protective factors in migraine and explain the role of oxidative stress in etiology.

## Materials and methods

### Study population

This case–control study included 148 patients with migraine who were recruited from the neurology department of the Henan Provincial People's and 150 age- and sex-matched outpatient health screeners derived from the physical examination center as healthy controls (HCs) between 2018 and 2021.

The diagnosis of migraine was determined by a neurologist using the International Classification of Headache Disorders-III (ICHD-III) diagnostic criteria (9). We divided the patients with migraine into subgroups for analysis according to ICHD-III clinical characteristics (episodic/chronic migraine;, attack/attack-free migraine, and migraine with aura/without aura) (10).

In terms of exclusion criteria, it was necessary to exclude patients with diseases associated with severe liver and kidney diseases, tumors, rheumatic immune diseases, acute or chronic infectious diseases, and endocrine system diseases that may affect antioxidant markers. Patients with history of neurological or psychiatric diseases, or combined with patent foramen ovale (PFO) are also excluded. Additionally, patients were excluded from the study if they have secondary headache.

The research protocol of this study was approved by the Research Ethics Committee of Henan Provincial People's Hospital, and all the participants signed written informed consent forms.

### Data collection and laboratory measurements

Self-reported demographic information included gender, age, frequency of headache attacks/month, duration of symptom attacks, presence of aura, the presence of phono-/photophobia, presence of nausea/vomiting, presence of autonomic manifestations, and development of resistance to medications. Additionally, their body mass index (BMI), which is defined as weight in kilograms divided by the square of height in meter, was directly measured (8). Heart rate, systolic blood pressure (SBP), and diastolic blood pressure (DBP) were measured with an electronic blood pressure monitor (OMRON, Japan).

All the research participants fasted overnight for 12 h, and venous blood samples were collected in a serum separation tube and centrifuged immediately according to standard operating procedures for examination of serum UA, ALB, CRE, TBIL, low-density lipoprotein cholesterol (LDL-C), high-density lipoprotein cholesterol (HDL-C), triglycerides (TGs), total cholesterol (TC), fasting blood-glucose (FBG), aspartate transaminase (AST), and alanine transaminase (ALT) levels established in this experiment. An ABBOTT Architect i2000SR automatic immune detection system (Abbott, United States) was used to measure these biochemical indicators.

### Migraine assessment

The severity of symptoms and sleep quality of the patients with migraine were assessed by professional physicians, including the Headache Impact Test-6 (HIT-6) (11), which measures the impact of migraine headache on daily activities, the Migraine Disability Assessment (MIDAS) (12, 13), which measures the severity of migraine, and the Pittsburgh Sleep Quality Index (PSQI) scale (14), which measures sleep quality.

### Statistical analysis

For data processing and statistical analysis, the SPSS Statics 21.0 computer software was utilized. Continuous variables are expressed as mean ± standard deviation (SD) or median and interquartile range (M, P_25_, P_75_) based on the normal or non-normal distribution of the data determined by Kolmogorov-Smirnov test. For normally distributed continuous data, we conducted Student's *t*-test, and for non-normally distributed continuous variables, we conducted Mann-Whitney U test. Chi-square test or Fisher's exact test was carried out to compare the groups. Categorical data are expressed in amount (%). After adjusting for the demographic and clinical data, a logistic regression analysis was performed to determine the independent predictors of migraine occurrence. Pearson or Spearman correlation coefficient (r) was used to describe the degree of relationship between antioxidant blood variables and clinical characteristics of migraine. The results were considered statistically significant when double-sided *p-*value was <0.05.

## Results

### Demographic and clinical characteristics

The biochemical and clinical characteristics of the participants are shown in Table 1. The serum levels of ALB, TBIL, CRE, and UA were significantly lower in the migraine group than in the HC group (*p* < 0.05). Additionally, for the levels of the other biochemical characteristics, there was no significant difference between both groups (Table 1). In the migraine group, 56 patients were in an attack period and 92 were in a free-attack period. The serum levels of ALB and UA were lower during attack periods than attack-free periods (*p* < 0.05). The serum levels of TBIL and CRE did not show significant differences (*p* > 0.05). There was no statistically significant difference between the blood antioxidant parameters investigated in the migraine with aura/migraine without aura and episodic/chronic migraine groups (*p* > 0.05) (Table 2).

**Table 1:** Clinical features and laboratory data of patients with migraine and controls.

**Variables**	**Migraine** **(*****n*** = **148)**	**Control** **(*****n*** = **150)**	***p*** **value**
Age (years)	40.59 ± 5.99	40.07 ± 5.82	0.447
Gender (male/female)	42/106	41/109	0.841
BMI (kg/m^2^)	23.42 ± 2.29	23.47 ± 2.49	0.869
SBP (mmHg)	126.89 ± 7.59	125.89 ± 6.07	0.208
DBP (mmHg)	79.18 ± 8.45	78.97 ± 8.35	0.825
ALB (g/L)	42.94 ± 3.01	44.39 ± 1.92	**<0.001**
TBIL (umol/L)	6.22 ± 1.09	7.08 ± 1.43	**<0.001**
FBG (mmol/L)	4.52 ± 0.42	4.57 ± 0.43	0.297
TC (mmol/L)	4.05 ± 0.78	4.11 ± 0.77	0.462
TG (mmol/L)	1.75 ± 0.86	1.76 ± 0.85	0.961
HDL-C (mmol/L)	1.03 ± 0.26	1.02 ± 0.25	0.734
LDL-C (mmol/L)	2.42 ± 0.72	2.47 ± 0.72	0.612
ALT (U/L)	28.33 ± 6.35	28.07 ± 8.94	0.766
AST (U/L)	23.21 ± 5.02	23.30 ± 4.73	0.884
ALP (U/L)	77.91 ± 9.45	76.89 ± 6.45	0.280
CRE (umol/L)	57.41 ± 8.51	59.23 ± 6.48	**0.038**
UA (ng/ml)	229.11 ± 15.91	233.85 ± 11.54	**0.004**

*BMI, body mass index; SBP, systolic blood pressure; DBP, diastolic blood pressure; ALT, alanine transaminase; AST, aspartate transaminase; FBG; fasting blood-glucose; TC, total cholesterol; TG, triglyceride; HDL-C, high-density lipoprotein cholesterol; LDL-C, low-density lipoprotein cholesterol; ALP, aspartate aminotransferase; ALB, albumin, TBIL, total bilirubin, CRE, creatinine, UA, uric acid. The bold values indicate the value of p < 0.05*.

**Table 2:** Serum levels of UA, TBIL, ALB, and CRE in migraine subtypes.

**Variables**	**Attack** **(*****n*** = **56)**	**Attack free** **(*****n*** = **92)**	***p*** **value**	**Episodic migraine** **(*****n*** = **81)**	**Chronic migraine** **(*****n*** = **67)**	***p*** **value**	**Migraine with aura** **(*****n*** = **55)**	**Migraine without aura** **(*****n*** = **93)**	***p*** **value**
ALB (g/L)	42.14 ± 2.51	43.42 ± 3.20	0.012	43.24 ± 2.38	42.57 ± 3.62	0.201	42.1 ± 2.51	42.82 ± 3.28	0.546
TBIL (umol/L)	6.17 ± 1.10	6.25 ± 1.09	0.677	6.33 ± 1.03	6.08 ± 1.15	0.185	6.23 ± 1.10	6.21 ± 1.09	0.899
CRE (umol/L)	59.09 ± 6.78	56.38 ± 9.30	0.060	228.38 ± 15.79	230.00 ± 16.14	0.540	228.58 ± 16.670	229.43 ± 15.53	0.791
UA (ng/ml)	222.98 ± 13.30	232.85 ± 16.27	<0.001	57.09 ± 9.17	57.79 ± 7.69	0.618	57.16 ± 8.85	57.55 ± 8.35	0.755

*ALB, albumin; TBIL, total bilirubin; CRE, creatinine; UA, uric acid*.

### Univariate and multiple stepwise logistic regression analysis

To identify the independent predictors, statistically significant variables from the univariate model were determined in the multiple stepwise logistic regression model, which ultimately reveled that ALB [odds ratio (OR) 0.79, 95% confidence interval (CI) 0.69–0.89, *p* < 0.001], TBIL (OR 0.61, 95% CI 0.5–0.75, *p* < 0.001), and UA (OR 0.97, 95% CI 0.96–0.99, *p* = 0.014) were independently associated with migraine (Table 3).

**Table 3:** Univariate and multiple logistic regression analyses to identify the predictors of migraine.

**Variables**	**Univariate**		**Multiple**	
	**OR (95%CI)**	***p*** **value**	**OR (95%CI)**	***p*** **value**
Age (years)	1.02 (0.98,1.06)	0.445	–	–
Gender (female/male)	1.05 (0.64,1.75)	0.841	–	–
BMI (kg/m^2^)	0.99 (0.90,1.09)	0.869	–	–
SBP (mmHg)	1.02 (0.99,1.06)	0.209	–	–
DBP (mmHg)	1.00 (0.98,1.03)	0.824	–	–
ALB (g/L)	0.77 (0.69,0.86)	**<0.001**	0.79 (0.69,0.89)	**<0.001**
TBIL (umol/L)	0.59 (0.49,0.72)	**<0.001**	0.61 (0.50,075)	**<0.001**
FBG (mmol/L)	0.75 (0.43,1.29)	0.297	–	–
TC (mmol/L)	0.90 (0.67,1.20)	0.461	–	
TG (mmol/L)	0.99 (0.76,1.30)	0.961	–	–
HDL-C (mmol/L)	1.17 (048,2.88)	0.733	–	–
LDL-C (mmol/L)	0.92 (0.67,1.27)	0.610	–	–
ALT (U/L)	1.00 (0.98,1.03)	0.766	–	–
AST (U/L)	0.99 (0.95,1.04)	0.884	–	–
ALP (U/L)	1.02 (0.99,1.05)	0.281	–	–
CRE (umol/L)	0.97 (0.94,0.99)	**0.040**	0.97(0.94,1.00)	0.105
UA (ng/ml)	0.98 (0.96,0.99)	**0.004**	0.97(0.96,0.99)	**0.014**

*BMI, body mass index; SBP, systolic blood pressure; DBP, diastolic blood pressure; ALT, alanine transaminase; AST, aspartate transaminase; FBG; fasting blood glucose; TC, total cholesterol; TG, triglyceride; HDL-C, high-density lipoprotein cholesterol; LDL-C, low-density lipoprotein cholesterol; ALP, aspartate aminotransferase; ALB, albumin; TBIL, total bilirubin; CRE, creatinine; UA, uric acid. The bold values indicate the value of p < 0.05*.

### Subgroup analysis by sex

The effects of sex on the serum levels of ALB, TBIL, CRE, and UA between the migraine and HC groups were determined. There were highly significant differences in the serum levels of ALB, TBIL, and UA between men and women in the migraine and HC groups (*p* < 0.05), which indicates a sex-specific difference (Table 4).

**Table 4:** Serum levels of ALB, TBIL, CRE, and UA in the migraine and HC groups.

**Patients**	**Male** **(*****n*** = **42)**	**Female** **(*****n*** = **106)**	**P** ^1^	**P** ^2^	**P** ^3^
**ALB**
Migraine	41.17 ± 2.34	43.64 ± 2.97			**0.000**
HC	44.03 ± 1.76	44.52 ± 1.98	**0.000**	**0.010**	0.162
**TBIL**
Migraine	6.27 ± 1.31	6.20 ± 0.99			0.715
HC	7.01 ± 1.66	7.11 ± 1.35	**0.028**	**0.000**	0.705
**CRE**
Migraine	60.33 ± 6.19	56.25 ± 9.04			**0.008**
HC	58.44 ± 5.99	59.53 ± 6.65	0.160	**0.003**	0.359
**UA**
Migraine	230.29 ± 11.35	228.65 ± 17.42			0.575
HC	235.68 ± 10.00	233.16 ± 12.04	**0.024**	**0.028**	0.233

*P^1^, male migraine patients vs. male HC; P^2^, female migraine patients vs. female HC; P^3^, male vs. female in each group. ALB, albumin; TBIL, total bilirubin; CRE, creatinine; UA, uric acid. The bold values indicate the value of p < 0.05*.

### Correlation between antioxidant blood variables and clinical characteristics of migraine

There was a statistically negative correlation between CRE serum level and HIT-6 scale (*r* = −0.178, *p* = 0.03) (Table 5).

**Table 5:** Correlation between antioxidant blood variables and clinical characteristics of migraine.

	**ABL**		**TBIL**		**CRE**		**UA**	
	***r***	***p***	***r***	***p***	***r***	***p***	***r***	***p***
HIT-6 scale	−0.062	0.453	−0.063	0.449	−0.178	0.030	−0.13	0.116
MIDAS	−0.107	0.222	−0.027	0.748	−0.046	0.579	0.048	0.560
Frequency of attacks/month	−0.021	0.794	−0.053	0.520	−0.017	0.836	−0.057	0.494
Attack duration	0.015	0.854	−0.047	0.570	−0.094	0.254	−0.031	0.711

*HIT-6, Headache Impact Test-6; MIDAS, Migraine Disability Assessment; ALB, albumin; TBIL, total bilirubin; CRE, creatinine; UA, uric acid. The bold values indicate the value of p < 0.05*.

## Discussion

In our study, we investigated the relationship among serum ALB, TBIL, CRE, and UA levels as indicators of oxidative stress to evaluate the antioxidant status of migraine. The results of this study showed that compared with the HC group, the serum levels of ALB, TBIL, CRE, and UA were significantly lower in the migraine group. In addition, ALB, TBIL, and UA were related to migraine. Moreover, there was a significant difference in the serum levels of ALB, TBIL, and UA between men and women in the migraine group on the sex subgroup analysis.

The pathomechanism of migraine is largely unknown. Systemic inflammation is known to be related to brain inflammation (15). Previous studies have shown increased peripheral inflammation in patients with migraine, which may contribute to the progression of the disease (16). Cytokines are directly involved in pain sensitization by acting both on peripheral nociceptive nerve terminals and sensory ganglia (17). A previous study has shown that high levels of oxidative stress are associated with migraine (18). Interestingly, a recent study on migraine with aura (MA) has revealed increased microglial activation the structures involved in pian processing including basal ganglia, the thalamus, insula, and somatosensory cortices, which provides insight into the role of neuroinflammation in migraine (19).

Previous studies showed altered mitochondrial enzyme activity in patients with migraine (20). Activation of nucleotide-binding domain (NOD)-like receptor family pyrin domain containing 3 (NLRP3) inflammasome by mitochondrial reactive oxygen species can cause hyperalgesia (21), which is involved in migraine pathophysiology. Neuroinflammation plays an important role in the pathophysiology of central nervous system diseases (22). We hypothesize that this finding may be due to scavenging of excess reactive oxygen species (ROS) by the antioxidant defense system of patients, ultimately leading to lower serum ALB, TBIL, CRE, and UA levels. ALB is capable of scavenging ROS and reactive nitrogen species (RNS) produced by various reactions in the body, and plays a major role as an antioxidant in the extracellular fluid (23). The results of our study found lower ALB levels in the patients with migraine. Although vomiting has been reported in patients with migraine, we do not believe that short-term vomiting causes hypoproteinemia in patients with migraine. We hypothesize that the strong state of oxidative stress is contributing to decrease in serum ALB. However, we did not show a difference between chronic and episodic migraines, which may be influenced by different patient characteristics as well as medication. TBIL is an endogenous scavenger of ROS (8). High oxidative stress during migraine may lead to lower serum TBIL concentration. CRE is a metabolite produced by muscle breakdown of creatine and is known to be an effective antioxidant event (24). In our study, CRE levels were lower in the patients with migraine than in the controls, reflecting lower antioxidant levels for migraine. UA is the final product of purine metabolism (25) and can scavenge reactive oxygen species in the body (26). Yazar et al. reported that UA levels were lower in patients with migraine (27), which was consistent with our findings. UA acts as an endogenous antioxidant that can reduce oxidative damage and inhibits inflammatory responses, which reduces the permeability of the blood-brain barrier (BBB) and exerts neuroprotective effects (28). Low serum UA levels have been defined as a risk factor for neurodegenerative diseases (29). However, it remains unclear whether UA is a protective factor or a risk factor for patients with migraine.

In this study, the serum levels of ALB, TBIL, CRE, and UA were significantly lower in the migraine group than in the HC group, indicating an association between oxidative stress and migraine. Several epidemiological studies on migraine have demonstrated that the prevalence of migraine in women is higher than in men (30). As shown in Table 4, by the sex subgroup analysis, the serum CRE and ALB levels of female patients were lower than those of male patients, suggesting weaker antioxidant capacity in women, which may provide a new perspective for explaining the higher incidence of migraine in women. However, the underlying mechanisms of changes in serum ABL, TBIL, CRE, and UA levels associated with patients with migraine are unclear and warrant clarification in future studies.

In recent years, more and more studies have confirmed that immunological dysfunctions can play an essential role in migraine pathophysiology (31). Previous studies found a positive association between migraine and several autoimmune disorders including systemic lupus erythematosus (32), subclinical hypothyroidism (33), and multiple sclerosis (34). Arumugam et al. found that the percentage of regulatory T cells (Treg cells) was significantly lower in patients with migraine (35). Faraji et al. also reported that Treg cells was decreased in patients with migraine in all subgroups (36). Treg cells can suppress inflammatory response and regulate the activity of immune system, and are a subset of CD4 + T cells (37). It was also found that significant decrease in the percentage of Treg cells has been considered to be a marker of autoimmune diseases (36). Growing evidence shows that there is a significant relationship between low serum ALB and TBIL levels and autoimmune diseases (38), which was consistent with our finding that decreased serum ALB and TBIL level was associated with migraine. Notwithstanding, further investigations are required to better elucidate the association between serum levels of ALB and TBIL and migraine.

Several limitations should be noted in our research. First, this was only a cross-sectional descriptive study and therefore lacked evidence of pathological mechanisms. Furthermore, prospective cohort studies are needed to clarify the mechanism of oxidative stress in migraine. Second, we did not analyze patients with migraine in subgroups of disease severity in our study. Finally, the number of patients studied was small, leading to possible type 1 errors and interesting trends that need to be confirmed in a larger series. Limited by sample size, the evidence to support the conclusion remains weak, thus further studies with larger populations are needed to define.

## Conclusion

Our study improves our understanding of the role played by oxidative stress in the pathogenesis of migraine. Serum levels of UA, TBIL, ALB, and CRE were reduced in the patients with migraine. In addition, reduced ALB, TBIL, and UA were independently associated with migraine. Furthermore, prospective cohort studies are warranted to determine the causality of these associations and to further clarify the mechanism of oxidative stress in migraine.

## Data availability statement

The original contributions presented in the study are included in the article/supplementary material, further inquiries can be directed to the corresponding author/s.

## Ethics statement

The studies involving human participants were reviewed and approved by the Research Ethics Committee of Henan Provincial People's Hospital. The patients/participants provided their written informed consent to participate in this study. Written informed consent was obtained from the individual(s) for the publication of any potentially identifiable images or data included in this article.

## Author contributions

ZZY: wrote first draft and statistics. ZZY and PFX: statistics and data collection. CFG and HJZ: conceptualization, resources, and supervision. All authors contributed to the article and approved the submitted version.

## Conflict of interest

The authors declare that the research was conducted in the absence of any commercial or financial relationships that could be construed as a potential conflict of interest.

## Publisher's note

All claims expressed in this article are solely those of the authors and do not necessarily represent those of their affiliated organizations, or those of the publisher, the editors and the reviewers. Any product that may be evaluated in this article, or claim that may be made by its manufacturer, is not guaranteed or endorsed by the publisher.
